# Cut-throat Injury in Attempted Suicide Amidst Coronavirus Disease 2019 (COVID-19)

**DOI:** 10.7759/cureus.49949

**Published:** 2023-12-05

**Authors:** Lee Kuang Joo, Nadiah Abdullah, Nor Shahida Abd Mutalib, Sakina Mohd Saad, Marina Mat Baki

**Affiliations:** 1 Department of Otorhinolaryngology and Head and Neck Surgery, Faculty of Medicine, Universiti Kebangsaan Malaysia Medical Centre, Kuala Lumpur, MYS; 2 Department of Psychiatry, Hospital Sultan Abdul Halim, Sungai Petani, MYS; 3 Department of Otorhinolaryngology and Head and Neck Surgery, Hospital Sultan Abdul Halim, Sungai Petani, MYS

**Keywords:** laryngeal injury, suicide, depression, movement-control order, covid-19

## Abstract

In the presence of the coronavirus disease 2019 (COVID-19) pandemic, a lengthy period of movement-control order had caused huge negative impact on the socioeconomic status of some patients and affected their mental health. Self-quarantine in this pandemic era serves as a major stressful event that may lead to psychosis and depression. Cases of suicide and attempted suicide raised drastically throughout the pandemic. We are discussing two cases of attempted suicide by people who were traumatized in different ways by the COVID-19 pandemic, but they chose the same solution with a similar method, cutting their throats in order to commit suicide. Both cases were proceeded with emergency neck exploration, laryngeal repair, and tracheostomy. Postoperatively, psychiatric and psychological treatment was initiated.

## Introduction

January 2020 marked the arrival of coronavirus disease 2019 (COVID-19) in Malaysia [1] where almost everyone was affected. Repeated lockdowns were implemented as cases and deaths related to COVID-19 started to escalate. One of the main consequences of the COVID-19 pandemic was the retrenchment and salary reduction of Malaysian workers. This was detrimental to people's financial well-being, as evidenced by Malaysia's unemployment rate which reached its highest level, 5.3%, in three decades in May 2020 [2]. Subsequently, these were affecting their mental health, as evidenced by the surge in recorded suicide cases. According to the Royal Malaysia Police (PDRM), the annual total of suicides in 2019 and 2020 were 609 and 631, respectively. A marked increase was noted in 2021 where 1,142 cases were recorded, almost 80% more compared to the number in the same period of time before COVID-19 [3]. COVID-19-related suicide is on the rise, but self-inflicted cutthroat is one of the rarer methods of suicide. Here, we discussed two cases of self-inflicted cutthroat which include the treatment plan that takes into account the extent of laryngeal injury as well as the patient's socioeconomic status.

## Case presentation

Case 1

**Figure 1:**
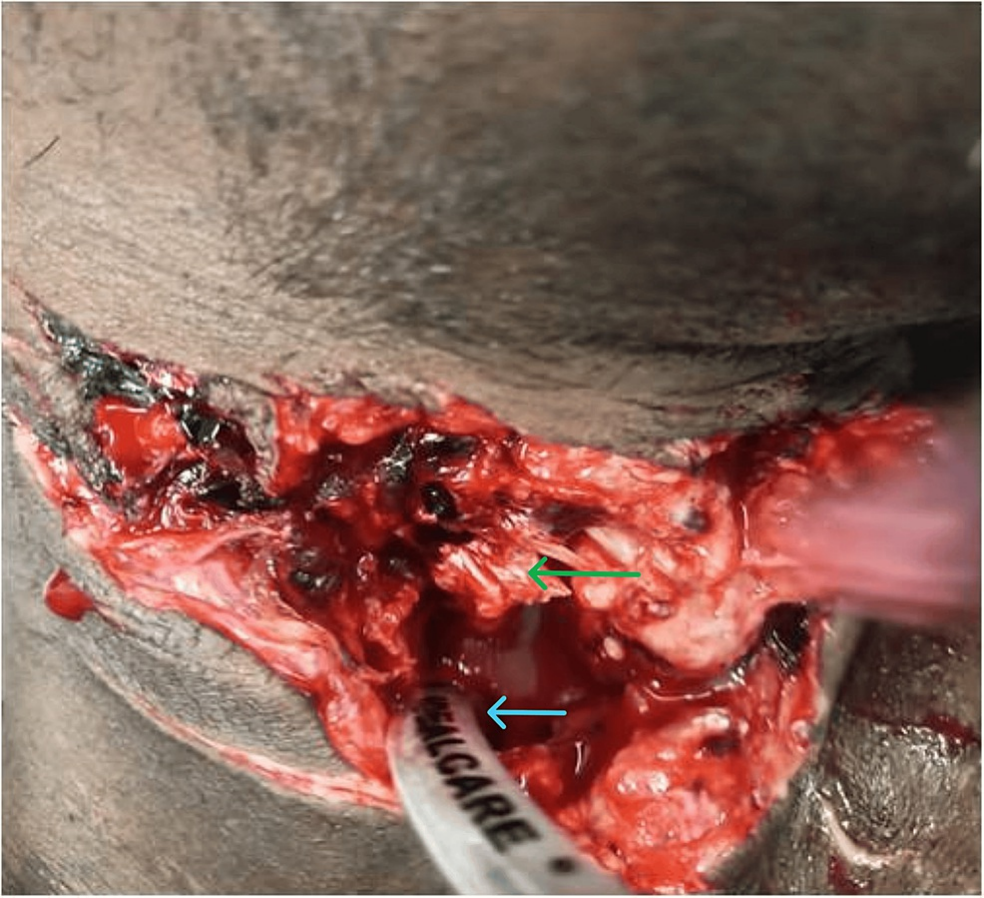
Endotracheal tube inserted through the open wound with the fractured anterior part of the tracheal rings. Green arrow: infrahyoid muscle. Blue arrow: fractured tracheal ring

Mister K is a 38-year-old male who has a history of intravenous drug abuse and is currently abusing methamphetamine. He has an underlying retroviral disease (RVD) and hepatitis C but was not receiving treatment. He was found lying unconscious outside an apartment with bleeding from an open neck wound secondary to suicide attempt in September 2021 and was brought to the emergency department (ED). His airway was successfully secured with an endotracheal tube inserted through an exposed tracheal cut by the emergency physician. Upon review by the otorhinolaryngology (ORL) team, it was discovered that he had suffered an extensive tracheal injury (Figure 1). The open neck wound was packed with gauze, and he was sent to the emergency operation theater for neck exploration and laryngeal repair. A segmented fracture of the lower half of the cricoid cartilage and a crashed fracture of the anterior portion of the first three upper tracheal rings with an intact trachealis muscle were discovered intraoperatively. The isthmus of the thyroid gland and infrahyoid muscle were also cut. There was no vascular or esophageal damage.

This was his second suicide attempt. The first attempt, two months prior, was driven by low mood and feeling helpless during the quarantine period while in close contact with a positive COVID-19 patient. He started experiencing auditory and visual hallucinations prior to the suicide attempt. He cut both of his wrists with a knife and sustained tendon-cut injury. A tendon repair procedure was performed, and he recovered well. Unfortunately, he became infected with COVID-19 (Cat 2), and this worsened his depression symptoms which led to his second suicide attempt. His urine tested positive for methamphetamine and opiates during his first hospitalization and positive for methamphetamine, amphetamine, and cannabis during his subsequent hospitalization. He was diagnosed with major depression with comorbid substance use disorder. He was started on antidepressant (fluvoxamine) and antipsychotic (olanzapine) medication.

**Figure 2:**
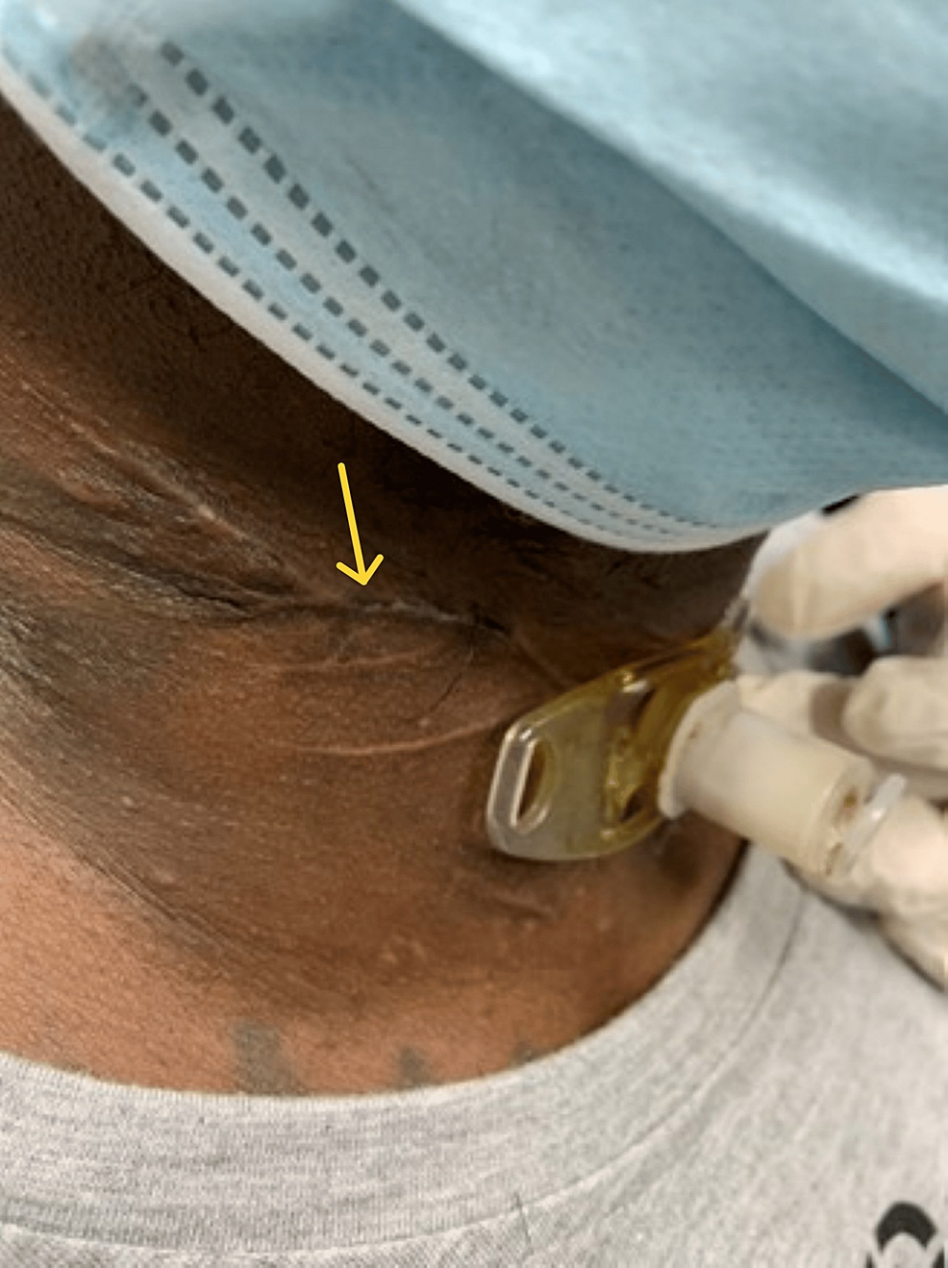
Postoperative one-month well-healed neck scar. Arrow: well-healed neck scar

**Figure 3:**
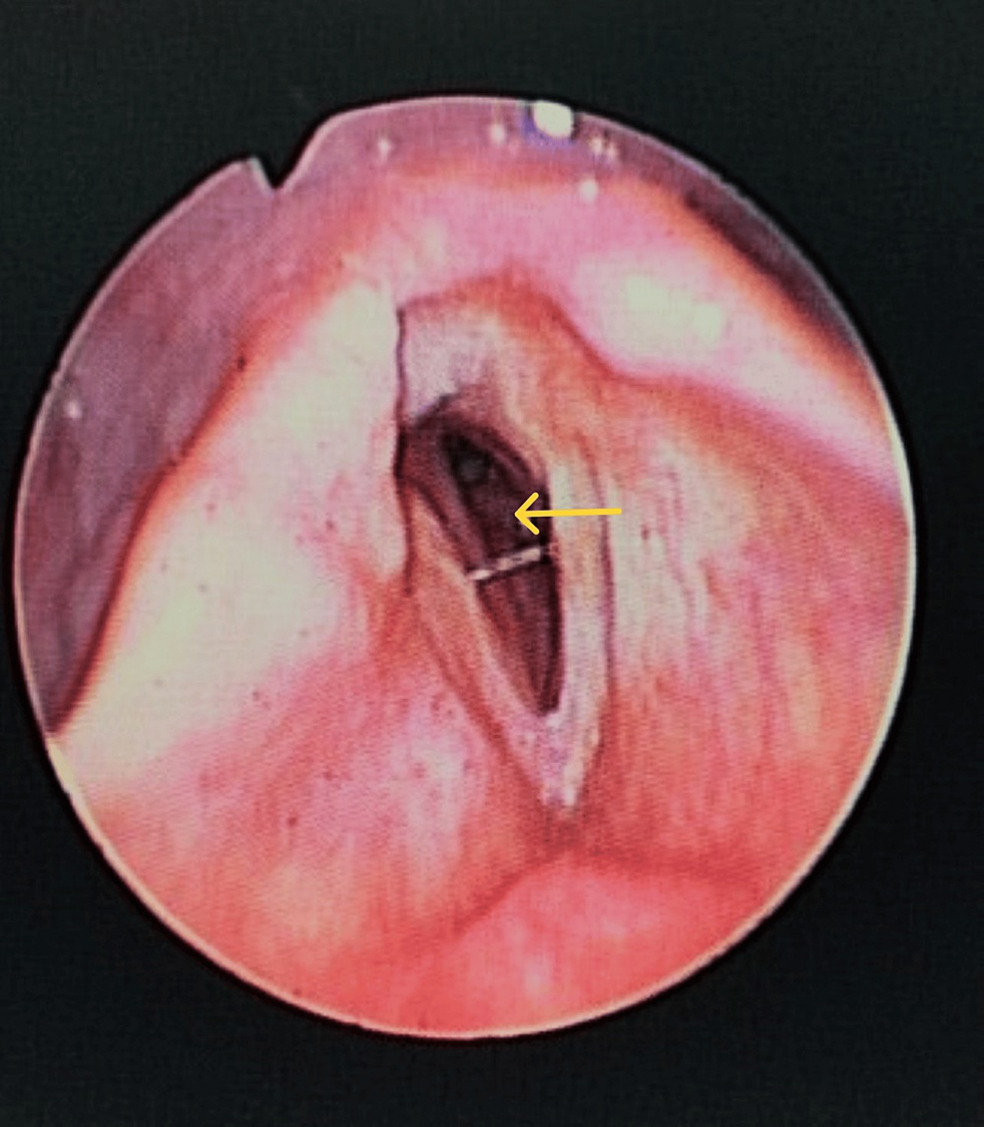
Postoperative one-month flexible laryngoscopy showed subglottic granulation tissue. Arrow: subglottic granulation tissue

Upon postoperative one-month review, his tracheostomy stoma and neck wound were clean and well healed (Figure 2). He was able to vocalize by capping his tracheostomy tube. However, the flexible laryngoscopy showed subglottic granulation tissue (Cotton-Myer grade 3) which cause narrowing of airway (Figure 3). Otherwise, his psychiatric symptoms were under control with the medication given. He refused for further operative intervention and opted for permanent tracheostomy with a double-lumen tracheostomy tube. He continued follow-up in ORL clinic for tracheostomy care for one-year duration but subsequently defaulted follow-up. 

Case 2

**Figure 4:**
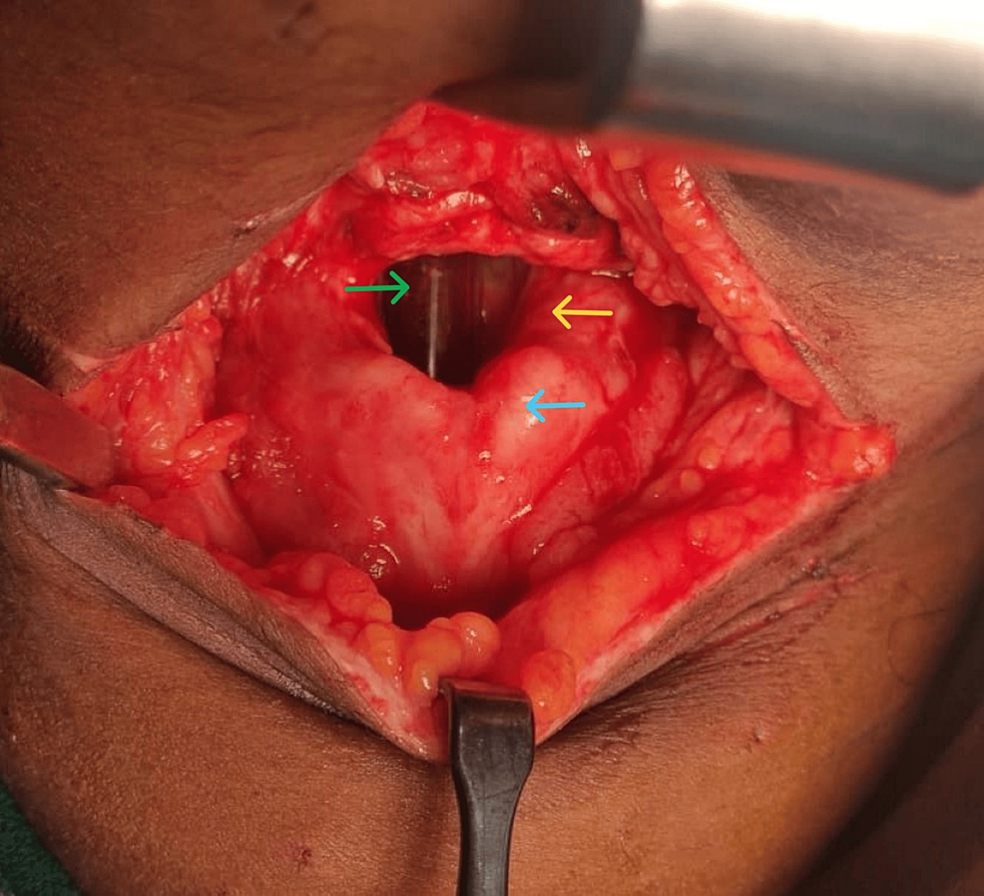
Horizontal neck wound cut through the thyrohyoid membrane. Green arrow: endotracheal tube. Yellow arrow: transected thyrohyoid membrane. Blue arrow: thyroid cartilage

Miss N is a 25-year-old female who worked as a quality control assessor at a local factory. She was referred to the ORL team for a neck injury following a suicide attempt at home by slashing her neck with a kitchen knife. In ED, oral intubation was performed by the emergency physician under video-assisted laryngoscopic guidance to secure her airway. Assessment revealed an irregular horizontal neck wound cut through the thyrohyoid membrane, readily exposing the intact thyroid cartilage and bilateral vocal folds through the neck wound (Figure 4). A computerized tomography (CT) scan revealed the trachea was intact without muscular or vascular damage. Neck exploration, tracheostomy, and direct laryngoscopy were done in an emergency setting.

One week prior, she was brought to ED for aggressive behavior and hallucinations. Unfortunately, her family refused hospital admission and opted for traditional treatment. With a history of sudden onset of psychosis, contrast-enhanced CT brain, magnetic resonance imaging (MRI) brain, and electroencephalogram (EEG) were performed during this hospitalization. However, there was no significant finding. Lumbar puncture was suggested but sadly declined. Further history revealed that she had been terminated from her job two weeks before her suicide attempt. Even though she was accepted at her current workplace, she felt insecure and pressured in trying to fit in the new environment. These major life-changing events caused her to have insomnia and reduced level of concentration and become easily fatigued.

From her psychiatric evaluation, she claimed that this is the first time she was hearing voices, few days before her suicide attempt, commanding her to harm her family members. Feeling distressed, she harmed herself instead. She was diagnosed with generalized anxiety disorder and treated with antidepressant (sertraline) and antipsychotic (olanzapine) medication. She was discharged well.

**Figure 5:**
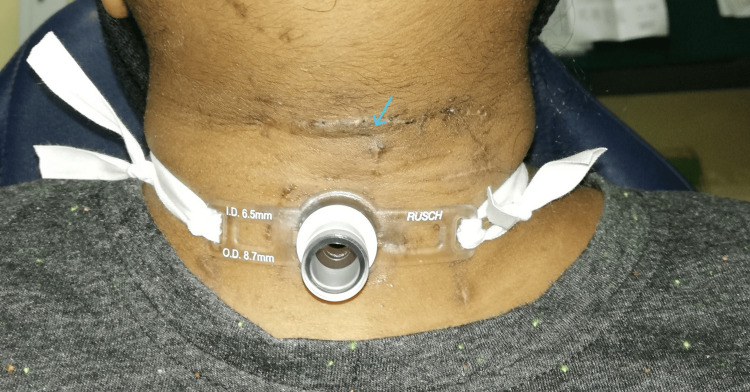
Postoperative one-month well-healed neck scar. Arrow: well-healed neck scar

Upon postoperative one-month review, her neck wound was well healed (Figure 5). She had a clean tracheostomy stoma and was emotionally stable. Flexible laryngoscopy revealed mobile and symmetrical bilateral vocal folds with normal laryngeal structures. Her tracheostomy tube was successfully decannulated at two-month postoperatively, and she is able to speak with normal quality of voice. Subsequent follow-up showed normal subglottic region, and she was discharged well.

## Discussion

The COVID-19 pandemic had great impact on mental health in the population worldwide including Malaysia. This exceptional situation has been causing social unrest which heightens anxiety and stress in people, thereby increasing the suicidal rate [4,5]. People who are isolated and stressed frequently turn to illegal substances to divert their negative feelings [6]. A study showed that more than 62% of the people maintained or worsened their consumption pattern during lockdown amidst the COVID-19 pandemic [7].

In our first case, Mister K is unemployed and a divorcee. He was abandoned by his family due to his drug addiction. He was noted to have symptoms of depression during his quarantine period. He was acting on an auditory hallucination commanding him to stab himself. The auditory hallucination was due to substance use rather than functional psychosis, as evidenced by his positive urine drug test. His first suicide attempt went unknown by his family members. The lack of family support received at that time pulled him deeper into severe depression, and the cycle repeated itself. The second time, he was using more substances, leading to his second suicide attempt with more severe injuries just two weeks after being discharged from the hospital. It was only after his second attempt that he was reunited with his family members. He stayed with his mother and sister after discharge. His depressive and psychotic symptoms had resolved and responded well to treatment.

The first case had severe laryngeal mucosa and cartilage disruption. The healing process leads to laryngotracheal stenosis. A series of surgical procedures will be needed to remove the granulation tissue and dilate the trachea. Eventually, tracheal resection and re-anastomosis might be required. However, the prognosis is guarded. His general health, underlying RVD status, mental well-being, and socio-family support need to be considered before proceeding with further reconstructive surgery. During the last review, the patient is not keen for further reconstructive surgery and is comfortable with his current mean of airway, tracheostomy tube.

As for the second case, she was one of those unfortunates affected by the cost-cutting measures adopted by her employer. Because of the COVID-19 pandemic, most businesses and factories were undergoing retrenchment, with many workers losing their jobs [7]. She was stressed and anxious as a result of losing her job and adjusting to a new work environment. Due to overwhelming stress and anxiety, she started to experience psychotic-like symptoms, resulting in suicidal attempt [8].

Contrary to the first case, the second case has a good prognosis in terms of the airway as the vocal fold and cartilages were intact and there was no subglottic involvement. We successfully decannulate her tracheostomy tube two months postoperatively. She is able to speak with normal quality of voice on decannulation and responding well to medication.

## Conclusions

Besides affecting one's health and socioeconomic status, the COVID-19 pandemic is also causing negative impact psychologically. Failure to cope with this stressful life event may lead to psychiatric symptoms and eventually contribute to suicidal attempts, as in our cases, self-inflicting cutthroat injury. In both of our cases, the prognosis depends on the severity of the laryngeal mucosa and cartilage involvement. These two cases highlight the importance of socio-family support during the COVID-19 pandemic at all levels. Postoperative care should include psychiatric and psychological treatment and socio-family support in addition to focus on the restoration of the airway and healing of the laryngeal injury.

## References

[1] (2023). Situasi Semasa Jangkitan Penyakit Coronavirus 2019 (COVID-19) Di Malaysia. https://www.moh.gov.my/moh/press_releases/KENYATAAN%20AKHBAR%20COVID19%202Mac2020.pdf.

[2] (2023). Labour Force, Malaysia, December 2020. https://v1.dosm.gov.my/v1/uploads/files/1_Articles_By_Themes/Labour_Force/Monthly/2020/Dis%2020/Labour%20Force%20Report%20December%202020.pdf.

[3] (2023). Tingkat Kesedaran, Tepis Stigma Terhadap Pesakit Mental. https://ukas.sarawak.gov.my/2022/10/11/tingkat-kesedaran-tepis-stigma-terhadap-pesakit-mental/.

[4] Kang JH, Lee SW, Ji JG, Yu JK, Jang YD, Kim SJ, Kim YW (2021). Changes in the pattern of suicide attempters visiting the emergency room after COVID-19 pandemic: an observational cross sectional study. BMC Psychiatry.

[5] Pietrabissa G, Simpson SG (2020). Psychological consequences of social isolation during COVID-19 outbreak. Front Psychol.

[6] Volkow ND (2020). Collision of the COVID-19 and addiction epidemics. Ann Intern Med.

[7] Jané-Llopis E, Anderson P, Segura L (2021). Mental ill-health during COVID-19 confinement. BMC Psychiatry.

[8] Galynker I, Ieronimo C, Perez-Acquino A, Lee Y, Winston A (1996). Panic attacks with psychotic features. J Clin Psychiatry.

